# Informative and Reliable Tract Segmentation for Preoperative Planning

**DOI:** 10.3389/fradi.2022.866974

**Published:** 2022-05-18

**Authors:** Oeslle Lucena, Pedro Borges, Jorge Cardoso, Keyoumars Ashkan, Rachel Sparks, Sebastien Ourselin

**Affiliations:** ^1^School of Biomedical Engineering and Imaging Sciences, King's College London, London, United Kingdom; ^2^Medical Physics and Biomedical Engineering, University College London, London, United Kingdom; ^3^King's College Hospital Foundation Trust, London, United Kingdom

**Keywords:** diffusion MRI, tract segmentation, deep learning, uncertainty quantification, calibration, tractography

## Abstract

Identifying white matter (WM) tracts to locate eloquent areas for preoperative surgical planning is a challenging task. Manual WM tract annotations are often used but they are time-consuming, suffer from inter- and intra-rater variability, and noise intrinsic to diffusion MRI may make manual interpretation difficult. As a result, in clinical practice direct electrical stimulation is necessary to precisely locate WM tracts during surgery. A measure of WM tract segmentation unreliability could be important to guide surgical planning and operations. In this study, we use deep learning to perform reliable tract segmentation in combination with uncertainty quantification to measure segmentation unreliability. We use a 3D U-Net to segment white matter tracts. We then estimate model and data uncertainty using test time dropout and test time augmentation, respectively. We use a volume-based calibration approach to compute representative predicted probabilities from the estimated uncertainties. In our findings, we obtain a Dice of ≈0.82 which is comparable to the state-of-the-art for multi-label segmentation and Hausdorff distance <10*mm*. We demonstrate a high positive correlation between volume variance and segmentation errors, which indicates a good measure of reliability for tract segmentation ad uncertainty estimation. Finally, we show that calibrated predicted volumes are more likely to encompass the ground truth segmentation volume than uncalibrated predicted volumes. This study is a step toward more informed and reliable WM tract segmentation for clinical decision-making.

## 1. Introduction

Segmentation of white matter (WM) tracts is important in several tasks including understanding brain organization, preoperative neurosurgical planning to identify eloquent areas, and identification of surgical approaches to reducing post-operative damage (1, 2). Clinically, manual tract annotations can help to plan a surgical approach but direct electrical stimulation is often used in complex cases as the ground truth to determine the precise location of eloquent areas during surgery (3). Manual tract annotations are time-consuming, often relying on fine-tuning tractography which depends on diffusion MRI (dMRI) acquisition parameters (4, 5), and suffer from inter- and intra-rater variability due to the complexity of the WM tracts (6–8). Automatic segmentation approaches have emerged to produce faster and more reproducible tract annotations. Nonetheless, to the best of our knowledge, none of the automatic approaches has investigated model reliability, such as uncertainty awareness, for tract segmentation.

Automatic tract segmentation methods can be divided into (1) region-of-interest-based (ROI) (or connectivity-based), (2) clustering-based, and (3) direct segmentation (8–10). ROI-based approaches focus on filtering tract fibers based on known anatomical regions before or after computing whole-brain tractography (11, 12). ROI-based methods require either brain parcellation or registration of subject-specific images to an atlas. Clustering-based methods focus on computing similarity metrics (i.e., distance) to classify WM fiber tracts (13–15). These approaches are computationally expensive due to the need to perform registration, whole-brain parcellation, or other preprocessing steps. Additionally, these methods demonstrate poor reproducibility in tracts that have high anatomical variability across subjects (16) which may introduce an unacceptable level of risk to the patient for preoperative neurosurgical planning.

Direct methods output tract masks or fiber tracts directly from input data without the intermediate steps of ROI-based or clustering-based methods (10). Direct methods can be divided into voxel-based or fiber-based classification approaches. Voxel-based methods classify voxels as being inside or outside a specific WM tract from volumetric input data while fiber-based methods classify whether or not a particular fiber belongs to a specific tract. Deep learning-based (DL) methods are currently the state-of-the-art for direct WM tract segmentation (8, 10, 17, 18). Once trained, DL models can quickly perform inference (19).

TractSeg (10), a voxel-based approach, uses 2D U-Nets (20), in a tri-planar approach, to segment 72 tracts. TractSeg uses as input the 3 major peak directions obtained from fiber orientation distributions (FODs) computed from constrained spherical deconvolution (CSD) (21). Neuro4Neuro (17), another voxel-based method, uses a 3D U-Net (22) to segment 25 tracts from an in-house dataset. As input for the 3D convolutional neural network (CNN), Neuro4Neuro uses diffusion tensor imaging (DTI) (23). DeepWMA (18), a fiber-based approach, uses a 2D CNN to classify fibers as belonging to one of 54 possible WM tracts. As input, DeepWMA uses a 2D multi-channel fiber feature descriptor computed for fibers obtained from whole-brain tractography. Similarly to DeepWMA, Classifyber (8) uses a set of features (i.e., spatial position, connectivity, etc) to describe a fiber of a tract and classify fibers into specific WM tracts. Classifyber uses a logistic regression (LR) model for classification.

Deep learning approaches tend to be overconfident in their segmentations which can lead to mistaken conclusions. For instance, underestimating the likelihood of a voxel being a false positive, missing a pathologic finding, or false negative leads to damaging an eloquent region (1, 24). Therefore, it is important to ensure model uncertainty is reflective of the ground truth data. Uncertainty estimation is also important as it enables DL results to be more transparent to the end-user giving clinicians more confidence in segmentation results.

Uncertainty quantification (UQ) can be used as a metric of reliability for DL approaches. UQ in DL has been investigated for a variety of medical imaging applications, such as physics-informed uncertainty-aware Brain MRI segmentation (25), modality synthesis (26), dMRI super resolution (27), brain parcellation (28), electrode bending prediction (29, 30), and tumor segmentation (24, 31, 32).

Uncertainty quantification is often divided into two types: epistemic, noise caused by variation in the model's parameters, or aleatoric, the noise inherent in the data (33). For tract segmentation, we expect the uncertainty to be both data and model-dependent. Thus, it is important to know where the model's parameters' variability causes uncertainty and where noise in the data causes uncertainty.

Epistemic uncertainty can be computed through Bayesian inference networks (BNNs) (33). BNNs offer a mathematically grounded method where they compute distribution functions for the trained parameters instead of regular scalars as in regular neural networks. However, they are hard to implement, and their training stage is computationally expensive (33). Bayesian approximation using dropout layers at the inference stage has been proposed to overcome training limitations of BNNs by doing multiple forward inferences (34) and has been successfully applied to medical imaging tasks (24, 27, 29). Following the Bayesian inference approximation, other methods such as Markov chain Monte Carlo (MCMC) (35) and Monte Carlo Batch Normalization (MCBN) (36) have been proposed where batch normalization at the inference stage approximates the outputs of a BNN.

Aleatoric uncertainty *via* learned loss attenuation, where a network is designed to have two branches one for the final prediction and one for uncertainty, has been proposed by Kendall and Gal (33). While this has been successfully applied to medical imaging tasks (27, 31, 37), the addition of a second branch makes the network challenging to train and prone to instability. Another method to compute aleatoric uncertainty is to augment input data at the inference stage and compute uncertainty over several rounds of inference. This approach is easy to implement once a CNN is trained and does not require modifying network architecture or retraining. Test time augmentation has been shown robust in medical imaging (32, 38).

An accurate segmentation model is important to achieve the best possible results and enable uncertainty to be generally low so that it highlights regions that are difficult to segment (either due to data or model limitations). However, the probability associated with the predicted class label does not always reflect its ground truth likelihood (39). Calibration makes predicted probabilities more aligned to the ground truth accuracy, meaning that output predictions reflect a measurable property in the annotations of a validation dataset. Calibration has been widely used as a post-processing step, e.g., in classification (40–42) and segmentation tasks (24, 43).

In this study, we aim to provide uncertainty awareness for tract segmentation with accurate and reliable predicted probabilities so that clinicians can use it as a safety tool in preoperative neurosurgical planning. We present a 3D CNN that takes as input raw dMRI intensities transformed into the spherical harmonics (SH) space to align data across subjects. We design a system to output calibrated epistemic and aleatoric uncertainties that are reflective of measured ground truth volumes. We demonstrate that our approach has comparable performance to the state-of-the-art tract segmentation approaches while providing an estimation of model and data uncertainty. The significance of this study is that it provides a method to augment information so clinicians can make more informed clinical choices.

## 2. Materials and Methods

### 2.1. Pipeline Overview

We project dMRI signal intensities into the SH space (Section 2.3) to align data across different acquisitions without fitting a model. Next, we train a 3D CNN to segment WM tracts from the SH coefficients (Section 2.3). Given a trained model, we calculate epistemic uncertainty (Section 2.7.1) and aleatoric uncertainty (Section 2.7.2). Finally, we perform volume-based calibration to make predicted probabilities and uncertainty measurements more representative of the ground truth volume (Section 2.8).

### 2.2. Dataset

We use dMRI from 105 subjects provided by the Human Connectome Project (HCP) (44). HCP dMRI were acquired on a 3T scanner with the following parameters: the spatial size of 145 × 174 × 145 with 1.25 mm isotropic resolution, 90 gradient directions for each b = ∈ {1,000, 2,000, 3,000 s/mm^2^} and 18 images at b = 0 s/mm^2^. Data is corrected following the protocols described in Sotiropoulos et al. (44) prior to download. For each one of the 105 HCP subjects, a set of 72 annotated tracts in the 3D spatial coordinate space, corrected by a human rater is provided by Wasserthal et al. (10) and available for download^1^. For each tract, a binary mask was generated similar to the approach of TractSeg (10). We set a voxel to the foreground if one or more fibers are present within the voxel.

### 2.3. Data Preprocessing

A single-shell (b = 2,000 s/mm^2^) was selected, and its dMRI signal intensities are transformed into SH coefficients without any model fitting. SH coefficients are then normalized by the b-zero shell using the algorithm provided in MRtrix (45). Then, we clamped all SH voxels outside the 5th and 99th intervals to remove outliers due to noise. In this study, we used *l*_*max*_ = 4 to compute SH coefficients as it has previously been demonstrated to provide comparable performance in CNN-based CSD model coefficient regression as *l*_*max*_ = 8 (46).

### 2.4. CNN Architecture

The CNN architecture used is the 3D U-Net (22) implementation provided with the nnU-Net framework presented in Isensee et al. (47). The nnU-Net implementation has four downsampling blocks in the encoder pathway and four upsampling blocks in the decoder pathway. Each downsampling block is comprised of 2 × (Convolution, Dropout, InstanceNorm, LeakyRelu) + pooling layer. The upsampling block has a similar structure but the pooling layer is replaced by an upsampling layer.

### 2.5. Data Augmentation

Classical techniques for on-the-fly augmentation including axis flipping, scaling, and rotation have been successfully applied to training DL models for small 3D medical imaging datasets (10, 48, 49). However, traditional medical image processing tools apply these augmentations to the 3D spatial domain which are inappropriate to apply to the SH domain. Therefore, to account for the SH coefficient properties, we apply the same random 3D rotation to both 3D spatial location and SH coefficients in order to ensure location and orientation are preserved during data augmentation as in Nath et al. (50).

### 2.6. CNN Training

For a given training dataset, let 𝕏 = [***X***_**1**_, …, ***X***_**τ**_] be the input images mapped to SH coefficients of order *l*_*max*_ = 4 and 𝕐 = [***Y***_**1**_, …, ***Y*****_τ_**] is the corresponding ground truth tract masks, where τ is the number of subjects. For a given pair of image ***X*****_τ_** = [***x*****_1_**, …, ***x*****_*J*_**], and mask ***Y*****_τ_** = [***y*****_1_**, …, ***y*****_*J*_]**, *J* is the number of voxels and ***x*****_*j*_** = [*x*_*j*1_, …, *x*_*jM*_], where *M* is the number of SH coefficients and ***y*****_*j*_** = [*y*_*j*1_, …, *y*_*jN*_], where *N* is the number of classes (tracts to be predicted). The training stage consists of optimizing the CNN model *f*_θ_(·) to minimize a mapping as follows:


(1) $$\begin{array}{l} \arg\underset{\theta }{\min}\left(f_{\theta }\left(𝕏,𝕐\right)\right) \end{array}$$

where ***θ*** are the learned weights. At the inference stage, for an image ***X*****_τ_**, we compute the predicted probabilities as $\hat{Y_{\tau }}=f_{\theta }\left(X_{\tau }\right)$.

#### 2.6.1. Loss Function

We used weighted binary cross-entropy (wBCE) loss during our CNN training stage. We calculate the distribution of each class over all subjects as ***C*** = [*c*_1_, …, *c*_*n*_] where $c_{n}=\overset{J}{\underset{j=1}{\sum }}y_{jn}$ is the number of positive labels for a given ground truth tract mask for a class *n* in the training set. A class weight $w_{n}=\frac{max\left(C\right)}{c_{n}}$ is used to preferentially optimize wBCE for classes with small numbers of positive labels. For a given predicted probability $\hat{Y_{\tau }}$ and ground truth tract masks ***Y*****_τ_**, we compute wBCE as:


(2) $$\begin{array}{l} wBCE(\hat{Y_{\tau }},Y_{\tau })=-\frac{1}{N}\sum_{n=1}^{N}w_{n}\sum_{j=1}^{J}(y_{jn}log({\hat{y}}_{jn}))\\ \;+\left(1-y_{jn})log(1-{\hat{y}}_{jn}\right)) \end{array}$$

#### 2.6.2. Training Setup

The 3D U-Net is initialized using the He uniform function (51) and is trained for 400 epochs, with a weight decay of 1*E* − 6, and a dropout of *r* = 0.25 in the encoder branch, based on experimentally chosen convergence. The learning rate is initialized to 1*E* − 3 and is reduced by 1/2 every 50 epochs. For each iteration in an epoch, a subject from the training set is randomly selected and 3D rotations (Section 2.5) are applied to augment the data in the range of [−20, 20]. From this image, 50 patches of size 64 × 64 × 64 × 15 are randomly sampled from within a binary mask corresponding to the intracranial space, where 15 is the number of SH coefficients. The number of patches was experimentally selected to achieve optimal convergence on the validation set while having all patches loaded on the available graphics processing units (GPUs). An epoch finishes when all subjects from the training set have been selected once. For every new subject, a new set of random rotations and patches are computed.

### 2.7. Uncertainty Quantification

Uncertainty can be divided into two types: epistemic (model's parameters' variability) or aleatoric (noise inherent in the data) (33). A well-established method for estimating epistemic uncertainty in deep learning is test time dropout (TTD) (34). In TTD, dropout layers are enabled at the inference stage to output multiple stochastic predictions. For aleatoric uncertainty, test time augmentation (TTA) is implemented by performing multiple data augmentations to the input data at the inference stage to output multiple stochastic predictions (32).

#### 2.7.1. Epistemic Uncertainty Modeling

We modeled epistemic uncertainty using TTD as described in Gal et al. (34). TTD estimates a tractable parametrized distribution q^*^(***θ***) which minimizes the Kullback-Leibler divergence of the true model posterior *p*(***θ***|𝕏, 𝕐) (33). However, *p*(***θ***|𝕏, 𝕐) is often not directly computable. In practice, without any further change in the model during the training, dropout layers that switch off neurons activation at a rate of *r*, sampled from a Bernoulli distribution, can be used at the inference stage to approximate probability distribution for the model weights ***θ***. This approximation is done by computing *T* forward passes where random model weights are set to 0 for each iteration. The final prediction is calculated by averaging the predicted probabilities from the *T* forward passes. The epistemic uncertainty is computed as the SD of the predicted probabilities from the *T* forward passes. In this study, we define a total of *T* = 20 forward passes and a dropout rate *r* = 0.25.

#### 2.7.2. Aleatoric Uncertainty Modeling

We modeled aleatoric uncertainty using TTA. This technique combines the predicted probabilities of multiple augmentation transforms at the inference stage to generate a final output to take into account noise inherent to the input data. TTA is common practice in classification problems in computer vision (52) and has also been applied in medical imaging for segmentation (32, 53). For TTA, the same data augmentation techniques as presented in Section 2.5 were used to augment the input data. Similarly to TTD, we define *T* = 20 forward passes, each with a random data augmentation.

#### 2.7.3. Aleatoric and Epistemic Uncertainty Modeling

Similarly to Wang et al. (32), we compute a Hybrid approach to compute both epistemic and aleatoric uncertainty using TTD and TTA, respectively. We keep *T* = 20 forward passes, where for each pass, we have a random data augmentation and a dropout rate *r* = 0.25. This gives a total of 20 predicted probabilities for each subject.

### 2.8. Calibration

We perform post-processing volumetric calibration as presented in Eaton-Rosen et al. (24). For a given subject ***x*****_*j*_**, after running *T* stochastic forward passes (TTD, TTA, or Hybrid), we have *T* predicted probabilities per voxel and per class ***ỹ***_*jn*_ = [ỹ_*jn*1_, …, ỹ_*jnT*_] for *n* ∈ [1, …, *N*] classes. We compute predicted probabilities quantiles ω_*kj*_ for each voxel given the *T* output predicted probabilities ***ỹ***_*jn*_ for $k\in \left[0,\frac{1}{T},\ldots ,\frac{T}{T}\right]$. Then, we compute the volume $V_{k}=\overset{J}{\underset{j=1}{\sum }}\omega_{kj}$ for each quantile *k*th. Next, the cumulative distribution function (CDF) *F*(*v*) = *P*(*V*_*k*_ < *v*) is computed where *V*_*k*_ is the kth quantile volume.

The calibration step is performed by fitting *F*(*v*) to a cumulative uniform distribution using a 1D linear interpolation. The scaling parameters for the linear interpolation are computed using a one-shot approach. This interpolation realigns *F*(*v*) so that the correct proportion of ground truth volumes appears in a given confidence interval (24). This calibration is performed per tract since each tract varies in shape, size, and uncertainty. We calculate all scaling parameters on the validation set, to prevent contamination with the test set, and subsequently use these trained parameters to calibrate predicted probabilities on the test set.

### 2.9. Evaluation Metrics

We evaluate the quality of predicted tract segmentation using the following metrics: sensitivity, specificity, Dice, Hausdorff distance, and average surface-to-surface distance (ASSD). Sensitivity is true positives over all predicted positive voxels, specificity is true negatives over all predictive negative voxels and Dice is the intersection of the predicted and ground truth masks over two times the union. The sensitivity, specificity, and Dice metrics are overlap metrics (larger numbers are best, the maximum value is 1.0). The Hausdorff distance measures the maximum of the directed distances between the boundaries of the predicted and ground truth segmentations, while ASSD is the average of all distances from points on the boundary of the predicted segmentation to the boundary of the ground truth segmentation and the boundary of the ground truth segmentation to boundary of the predicted segmentation (54). ASSD and Hausdorff distance measures often indicate if outliers are present in the predicted segmentation (55). The Hausdorff distance and ASSD are distance-based metrics (smaller numbers are best, the minimum value is 0.0).

### 2.10. Experiments

We assess the following tract segmentation approaches, deterministic (U-Net) and stochastic (TTD, TTA, and Hybrid), in the following scenarios: (1) how well the deterministic and stochastic approaches perform tract segmentation (*Segmentation performance and comparison to state-of-the-art*), (2) how well the deterministic and Hybrid approaches perform on clinical quality data (*Segmentation performance on clinical quality data*), (3) how well uncertainty maps computed by the stochastic approaches correlate with tract segmentation error (*Correlation between uncertainty and segmentation error*), and (4) how well volume-based calibration adjusts predicted probabilities from TTD, TTA, and Hybrid approaches (*Calibration impact on predicted tract volume*). The details of each experiment are described below.

#### 2.10.1. Segmentation Performance and Comparison to State-of-the-Art

We assess how well deterministic and stochastic tract segmentation approaches perform in terms of the evaluation metrics described in Section 2.9. In this experiment, 5-fold cross-validation is conducted where 4 folds are used for training (10% of training data was used for validation) and 1 fold for testing. We also compare the results of our approaches against state-of-the-art approaches, including TractSeg (10) (CNN-based method), Classifyber (8) (machine learning-based method), and RecoBundles (56) (a clustering-based method for segmentation) in terms of average Dice.

#### 2.10.2. Segmentation Performance on Clinical Quality Data

We assess the robustness of the deterministic and hybrid approaches perform on a clinical quality dataset. From the original HCP data, we first select the b = 1,000 s/mm^2^ shell to mimic a shell commonly acquired in a clinical protocol. Then, similarly to Lucena et al. (46), for each dataset, we first reorder the set of gradient directions such that if a scan is truncated the acquired gradient directions will still be close to optimally distributed on the half-sphere (45), and we then synthetically generate a clinical quality dMRI scan by truncating the number of gradient directions for b = 0 and b = 1,000 s/mm^2^ to 45 gradient directions. Finally, we apply the method described in this article (Section 2).

#### 2.10.3. Correlation Between Uncertainty and Segmentation Error

We assess how well uncertainty quantification correlates with tract segmentation errors. We use structure-wise uncertainty measured by the volume variation coefficient (VVC) and correlation this to segmentation errors as measured by 1 - average Dice as presented by Wang et al. (32). We compute Dice for each of *T* forward passes and then compute the average of the *T* Dice scores (output predict probabilities images are thresholded at ≥0.5 to obtain a binary segmentation). We compute tract volume for each forward pass, *V* = [*v*_1_, ...*v*_*T*_] where *v*_*t*_ is the total sum over all voxels on the binary image and *t* ∈ [0, …, *T*]. VVC is then computed as *VVC* = σ_*V*_/μ_*V*_ where μ_*V*_ and σ_*V*_ are the mean and SD for all volumes in *V*, respectively. We compute the strength of the VVC and 1 - Dice correlation using Spearman's rank correlation coefficient (57). Spearmans' correlation assesses monotonic relationships (whether linear or not). If there are no repeated data values, a perfect Spearmans' correlation of 1 or −1 occurs when each of the variables is a perfect monotone function of the other (57).

#### 2.10.4. Calibration Impact on Predicted Tract Volume

We assess how well volume-calibrated stochastic methods (TTA, TTD, and Hybrid) correspond to ground truth volumes. In this experiment, we evaluate the correlation of predicted volumes at different quantiles obtained over *T* forward passes, with and without calibration, to the ground truth volumes for individual tract structures.

### 2.11. Implementation

All experiments were performed on a workstation equipped with an Intel CPU (Xeon®W-2123, 8 × 3.60 GHz; Intel), 32 GB of memory, and an NVIDIA GPU (GeForce Titan V) with 12 GB of on-board memory. All code was implemented in Python 3.6. PyTorch 1.6.0 (58) and PyTorch lightning (59) were used for network training. MONAI 0.5.2 and TorchIO 0.18.15 (60) were used for data loading and sampling. Data augmentation was performed using SHtools 4.6.2 (61). All code used for training the models is available online as an open-source project^2^.

## 3. Results

**Figure 1:**
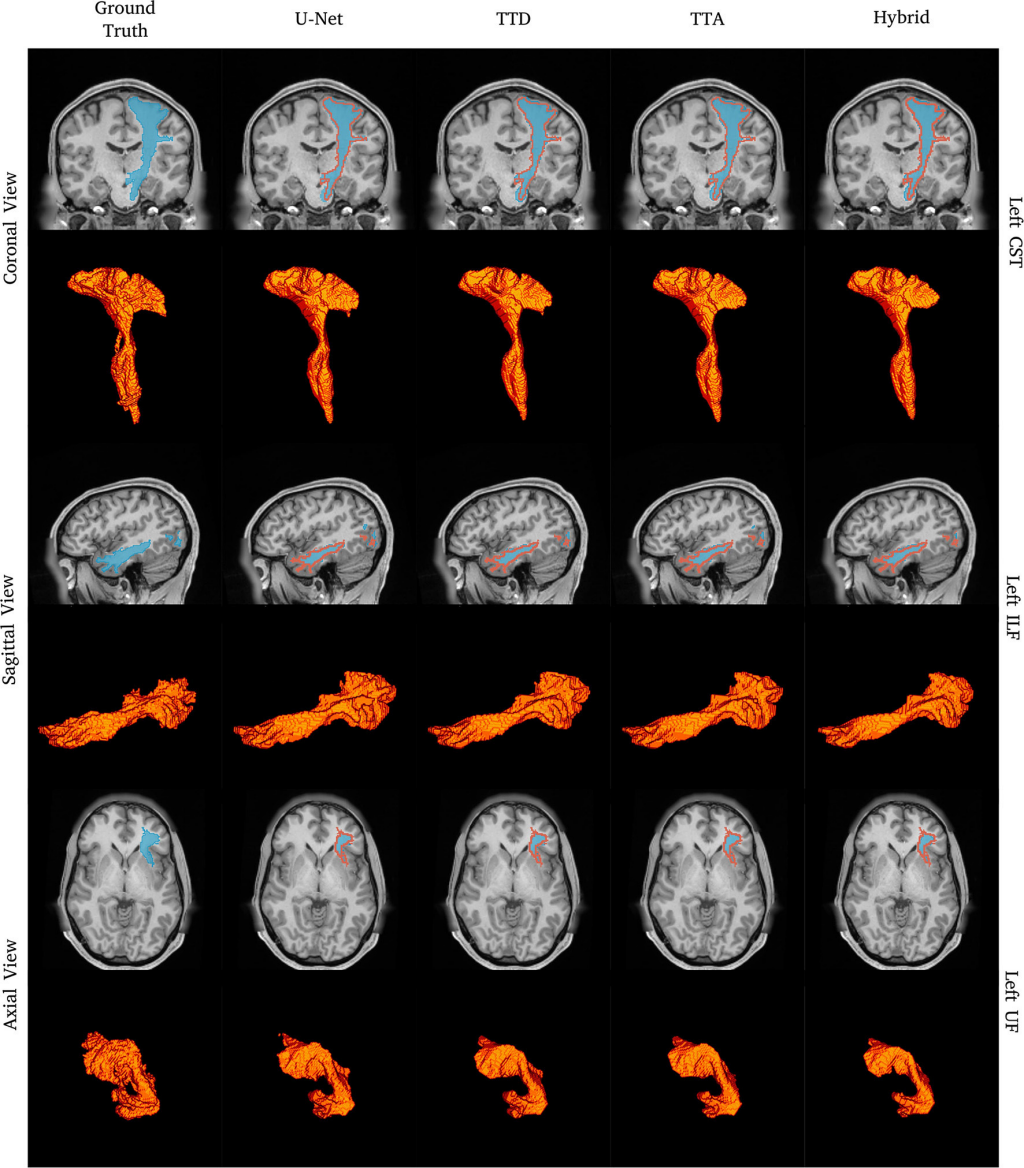
Tract segmentation comparisons between deterministic and stochastic approaches for the left CST, ILF, and UF tracts. Red contours show ground truth segmentations.

**Figure 2:**
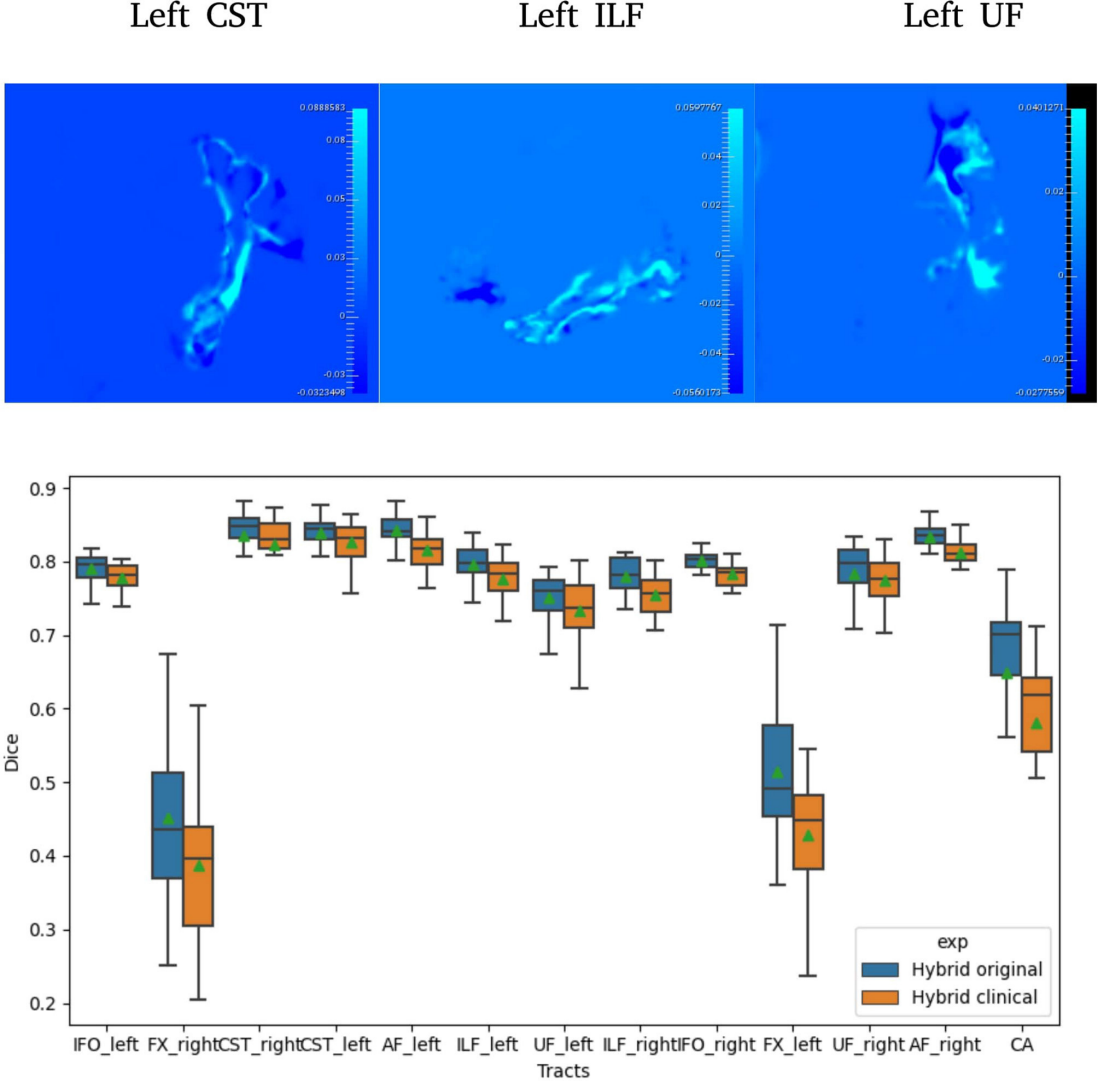
Difference between Hybrid the SDs of the predicted probabilities (Uncertainties) computed on original and clinical data (top). A selection of Dice distribution for 13 select tracts is computed across all 105 subjects (bottom).

**Figure 3:**
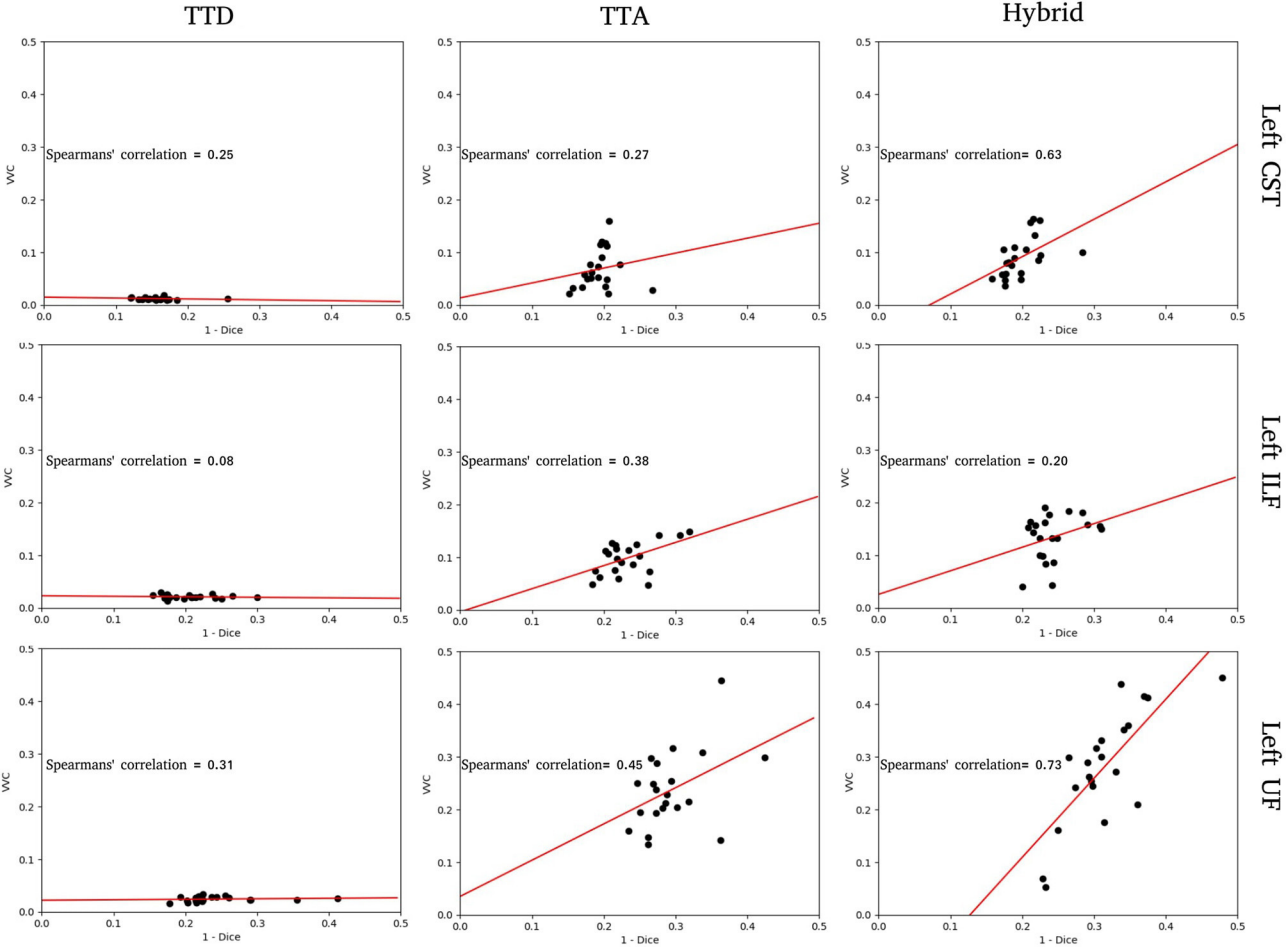
Structure-wise uncertainty as measured by volume variation coefficient (VVC) vs. 1 - Dice for TTD, TTA, and Hybrid approaches in the left CST, ILF, and UF tracts. Spearman's correlation coefficient indicates the strength of the correlation between VVC and 1 - Dice.

**Figure 4:**
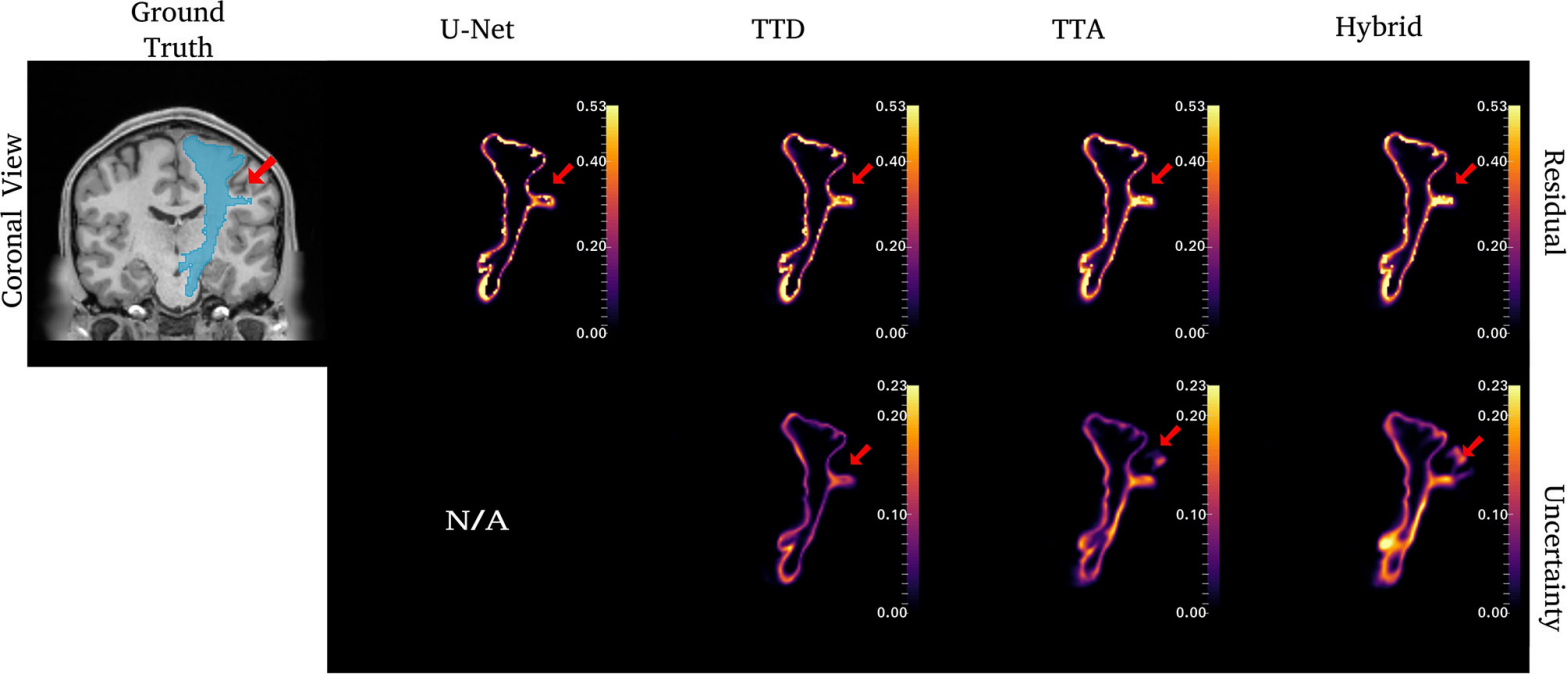
Uncertainty and residuals maps for the left CST tract. Red arrows point to one area of high uncertainty within the ground truth. This region may not represent real CST anatomy. Uncertainty maps are not applicable (N/A) for the deterministic U-Net approach.

**Figure 5:**
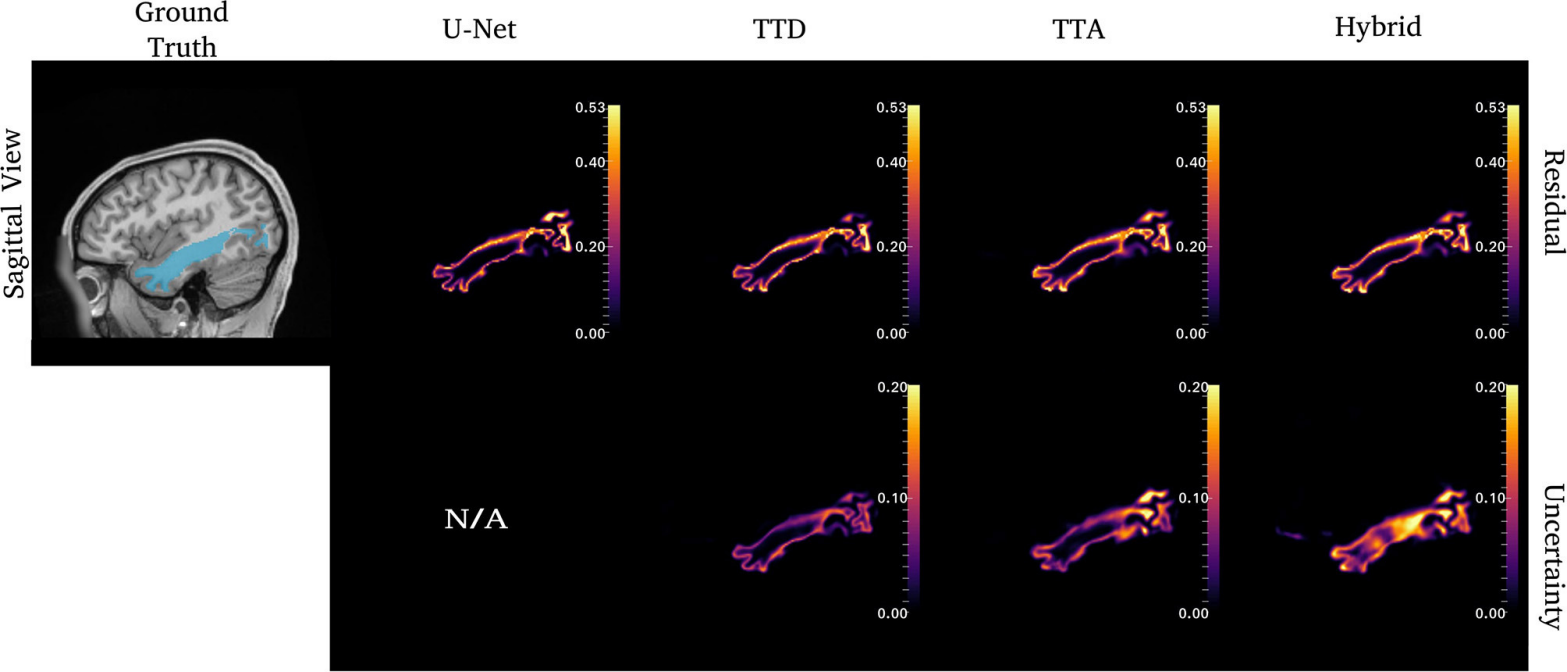
Uncertainty and residual maps for the left ILF tract. Uncertainty maps are not applicable (N/A) for the deterministic U-Net approach.

**Figure 6:**
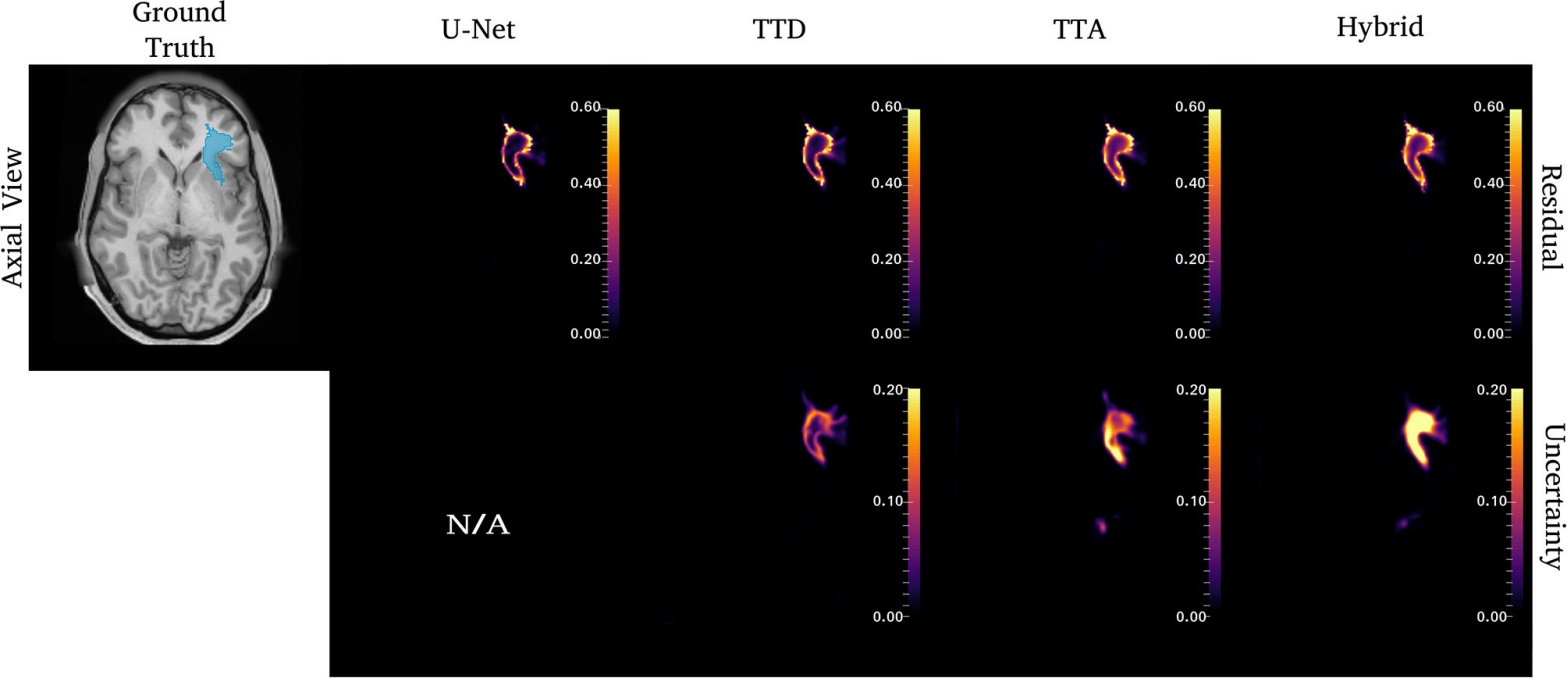
Uncertainty and residual maps for the left UF tract. Uncertainty maps are not applicable (N/A) for the deterministic U-Net approach.

**Figure 7:**
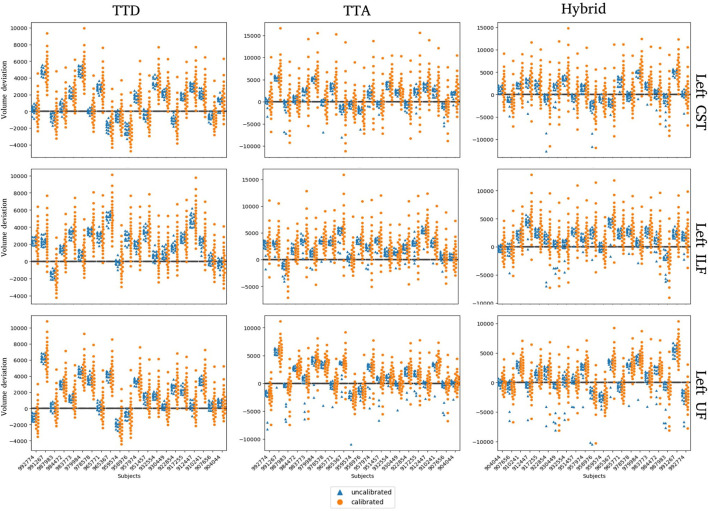
Uncalibrated and calibrated predicted volumes for TTD, TTA, and Hybrid approaches. Volume deviation is computed as the predicted volume subtracted by the ground truth volume. For each subject, the ground truth (black horizontal line) is at zero, and the predicted volume deviation is more visually apparent.

Overall quantitative analyses are reported for all 72 tracts (Tables 1, **4**) while other detailed results for specific qualitative and quantitative are presented for a small number of select tracts (Table 2 and Figures 1–**7**). A complete list of all tracts can be found online^1^. For these cases, we chose to report the representative tracts: corticospinal tract (CST), inferior longitudinal fascicle (ILF), and uncinate fascicle tract (UF) for the left side of the brain. CST is a large, well-represented tract with a straight shape that fans out close to the cortex. ILF is a complex longitudinal tract that starts from the anterior side and goes to the posterior side of the brain. Finally, the UF is a complex tract that has a large “C” shaped curvature.

**Table 1:** Dice, sensitivity, specificity, ASSD, and Hausdorff distance evaluation for deterministic (U-Net) and stochastic (TTD, TTA, and Hybrid) approaches.

**Network^a^**	**Dice**	**Sensitivity**	**Specificity**	**ASSD (mm)**	**Hausdorff distance (mm)**
TractSeg^a^	**0.84**	-	-	-	-
U-Net	0.83 (0.06)	0.81 (0.09)	**0.85 (0.07)**	0.69 (0.63)	17.32 (21.66)
TTD	0.82 (0.06)	0.85 (0.08)	0.80 (0.08)	0.65(0.28)	10.57 (9.47)
TTA	0.82 (0.07)	0.85 (0.08)	0.80 (0.09)	**0.63 (0.30)**	**9.24 (3.73)**
Hybrid	0.82 (0.07)	**0.86 (0.08)**	0.78 (0.09)	0.66 (0.33)	9.46 (3.74)

a *TractSeg results are taken from Wasserthal et al. (10). The best value, the minimum value for ASSD and Hausdorff distance, and the maximum value for Dice, sensitivity, and specificity, are indicated by bold text*.

**Table 2:** Comparison with state-of-the-art approaches for the following 5 tracts from both left and right sides of the brain: arcuate fascicle (AF), corticospinal tract (CST), inferior fronto-occipital fascicle (IFO), inferior longitudinal fascicle (ILF), and uncinate fascicle (UF).

**Tract**	**RecoBundles^**a**^**	**TractSeg^**b**^**	**Classifyber^**c**^**	**U-Net**	**TTD**	**TTA**	**Hybrid**
Right CST	0.62	0.85	0.87	0.84 (0.03)	0.84 (0.03)	0.84 (0.03)	0.84 (0.03)
Left CST	0.62	0.85	0.86	0.85 (0.03)	0.85 (0.02)	0.85 (0.02)	0.84 (0.02)
Right UF	0.57	0.79	0.86	0.77 (0.04)	0.78 (0.03)	0.78 (0.03)	0.78 (0.03)
Left UF	0.55	0.77	0.84	0.75 (0.07)	0.75 (0.06)	0.76 (0.06)	0.75 (0.06)
Right AF	0.53	0.83	0.86	0.83 (0.03)	0.83 (0.02)	0.84 (0.02)	0.83 (0.02)
Left AF	0.71	0.84	0.83	0.84 (0.03)	0.84 (0.02)	0.85 (0.02)	0.84 (0.02)
Right ILF	0.42	0.75	0.82	0.80 (0.03)	0.79 (0.02)	0.81 (0.02)	0.80 (0.02)
Left ILF	0.57	0.77	0.84	0.80 (0.03)	0.79 (0.02)	0.80 (0.03)	0.79 (0.02)
Right IFO	0.76	0.80	0.84	0.80 (0.04)	0.80 (0.03)	0.80 (0.04)	0.79 (0.03)
Left IFO	0.67	0.80	0.84	0.78 (0.04)	0.78 (0.03)	0.78 (0.03)	0.78 (0.03)

a *Garyfallidis et al. (56)*.

b *Wasserthal et al. (10)*.

c *Bertò et al. (8)*.

*Results for TractSeg, ReconBundles, and Classifyber are reported in Bertò et al. (8)*.

### 3.1. Segmentation Performance and Comparison to State-of-the-Art

Table 1 reports the mean (standard deviation) for the metrics described in Section 2.9 for both deterministic (U-Net) and stochastic (TTA, TTD, Hybrid) approaches. Similar performance in terms of Dice is found between U-Net and TTD, TTA, and Hybrid (average Dice ≈0.82). Stochastic approaches are more sensitive than the deterministic approach but less specific, which can be explained due to the presence of fewer false negative predictions (segmenting a voxel that belongs to a specific tract as background) by the TTD, TTA, and Hybrid approaches.

Pronounced improvements in Hausdorff distance are found for stochastic approaches compared to the deterministic approach (for TTA, it is a ≈50% improvement, 9 mm difference). This demonstrates that stochastic approaches are less likely to make large mistakes when segmenting a tract. TTD and TTA are comparable in terms of Dice performance to TractSeg which currently has the best achieving results for multi-label tract segmentation.

Table 2 compares the Dice performance between our approaches and four state-of-the-art tract segmentation methods. Both deterministic and stochastic approaches have comparable performance to TractSeg as expected from the results in Table 1. RecoBundles, an ROI-based method, has the lowest Dice performance while Classifyber, an LR fiber-based classification approach has the highest Dice performance. However, differences in Dice are relatively small (1–7%) between methods. ReconBundles is known to demonstrate poor reproducibility in tracts with high anatomical variability across subjects (16) while Classifyber relies on LR where each tract is treated as a separated classification task and does not face the challenges of multi-label classification.

Figure 1 shows qualitative segmentations for CST, ILF, and UF on the left side of the brain. Both deterministic and stochastic approaches provide tract segmentation that has similar shapes and sizes compared to the ground truth tract masks. For the UF, there is larger anatomical variability and fewer “spurious” regions in the inner part of the tract resulting in a cleaner “C” shape, when compared to the ground truth. This can be explained due to CNN's learning an average pattern across different subjects during the training stage leading to smoother results.

### 3.2. Segmentation Performance on Clinical Quality Data

Table 3 reports the mean (SD) for the metrics described in Section 2.9 for both U-Net and Hybrid approaches evaluated on the clinical data. As expected, due to the lower quality of the clinical data and different acquisition parameters, we observe a drop in performance on clinical data (45 gradient directions, b = 1,000 s/mm^2^) for both U-Net and Hybrid approaches when compared to the original data (90 gradient directions, b = 2,000 s/mm^2^). For the Hybrid approach, we observe greater uncertainty in the boundary regions resulting in under segmentation of the tract (Figure 2). This trend is observed across all tracts, with a drop in performance of 4%. These results highlight the importance of computing uncertainty for lower quality data in preoperative planning.

**Table 3:** Dice, sensitivity, specificity, ASSD, and Hausdorff distance evaluation for deterministic (U-Net) and stochastic Hybrid approaches for clinical quality data.

**Network**	**Dice**	**Sensitivity**	**Specificity**	**ASSD (mm)**	**Hausdorff distance (mm)**
U-Net	0.82 (0.06)	0.83 (0.09)	0.81 (0.07)	0.68 (0.55)	16.18 (20.84)
Hybrid	0.78 (0.08)	0.89 (0.08)	0.72 (0.11)	0.81 (0.44)	10.20 (3.90)

### 3.3. Correlation Between Uncertainty and Segmentation Error

Figure 3 plots VVC (tract uncertainty) vs. 1-Dice (segmentation error). A high correlation between VCC and 1-Dice is an indication of segmentation reliability, whereas uncertainty is indicative of poor segmentation performance (i.e., higher error). Therefore, a positive correlation should be expected if the uncertainty computed is a good measure of segmentation reliability.

For the tracts segmented by the TTD approach, the correlation between structure-wise uncertainty and segmentation error is absent (ILF) or weak (CST, UF). This indicates that model uncertainty is an inadequate measure of segmentation reliability. The TTA approach had stronger correlations between structure-wise uncertainty and segmented error. This demonstrates that aleatoric uncertainty provides a good measure of segmentation reliability in this task. For the tracts segmented by the Hybrid approach, the strongest correlations between structure-wise uncertainty and segmentation error are observed, suggesting that including both model and data uncertainty is more beneficial to estimating the reliability of the segmentation than either measure individually. For these specific tracts, for all stochastic approaches, UF and ILF exhibit higher slopes compared to CST which can be explained due to these structures having more complex tract anatomy.

Figures 4–**6** show 2D reconstructions of the residuals (***y*** − ***ŷ***) for U-Net, TTD, TTA, and Hybrid approaches and uncertainty maps output by the stochastic approaches. For all tracts, residuals tend to be at boundary voxels (regions more likely to be mistaken for other tracts) and these regions are also associated with higher uncertainty (TTD, TTA, and Hybrid only).

For the CST (Figure 4), stochastic approaches have larger residuals in areas that may not be part of the tract (small protuberance on the right side of the CST with high uncertainty). TTD demonstrates high uncertainty for areas with high residuals values whereas TTA and Hybrid approaches identify uncertainty in more dispersed regions, although these approaches still have high uncertainty near boundary regions. This may occur due to variability in the tract shape caused by multiple augmentations.

Similar to CST (Figure 5), for the ILF, TTD uncertainty is highest at boundary voxels while TTA and Hybrid also output high uncertainty for regions inside the tract. For this case, the Hybrid approach outputs high uncertainty in many areas inside the tract which may be due to the complex tract structure. For the UF, a tract with a very high curvature that varies between subjects, TTA residuals output high values inside the tract similarly to the Hybrid approach (Figure 6).

### 3.4. Calibration Impact on Predicted Tract Volume

We evaluated how well volume-based calibration for the stochastic approaches impacts the reliability of the predicted probabilities. Table 4 reports the mean (SD) for all metrics described in Section 2.9 for uncalibrated and calibrated approaches. As expected, no pronounced difference is found within the segmentation metrics between the uncalibrated and calibrated approaches.

**Table 4:** Segmentation metrics for 21 random subjects in the test set.

**Network**	**Dice**	**Sensitivity**	**Specificity**	**ASSD (mm)**	**Hausdorff distance (mm)**	**Calibration**
TTD	0.81 (0.07)	0.84 (0.08)	**0.81 (0.09)**	0.67 (0.31)	10.75 (9.82)	NO
TTD	0.81 (0.07)	0.84 (0.08)	**0.81 (0.09)**	0.67 (0.32)	10.78 (10.04)	YES
TTA	0.82 (0.08)	0.84 (0.09)	0.80 (0.10)	0.67 (0.38)	9.49(4.01)	NO
TTA	**0.83 (0.05)**	0.84 (0.08)	0.81 (0.10)	**0.67 (0.22)**	**9.48 (4.04)**	YES
Hybrid	0.81 (0.08)	**0.86 (0.08)**	0.79 (0.11)	0.69 (0.4)	9.65 (3.86)	NO
Hybrid	0.81 (0.08)	**0.86 (0.08)**	0.78 (0.11)	0.70 (0.42)	9.70 (3.90)	YES

*In this table, TTD, TTD, and Hybrid results are computed from uncalibrated and calibrated predicted probabilities. The best value, minimum value for ASSD and Hausdorff distance, and maximum value for Dice, sensitivity, and specificity, are indicated by bold text*.

Volume-based calibration makes the distribution of predicted volumes more uniform to consequently make predicted probabilities more representative of the ground truth volume. Calibrated predicted volumes (orange dots) tend to encompass the ground truth segmentation volume (black bars) compared to uncalibrated predicted volumes (blue dots) (Figure 7).

For all 105 subjects, we compute the minimum volume difference between the ground truth volume and the predicted volume for the Hybrid approach before and after calibration (Table 5). The average minimum volume difference between ground truth volume and calibrated predicted volumes is lower than the average minimum volume difference between ground truth volumes and uncalibrated predicted volumes. These results indicate that calibration makes the distribution of predicted volumes more representative of the ground truth volumes for the dataset.

**Table 5:** The minimum volume difference between ground truth volume and the predicted volume for the Hybrid method before and after calibration.

**Tracts**	**Minimum volume difference**	
	**Calibrated**	**Uncalibrated**
Left CST	347.83 (444.96)	90.82 (50.87)
Left ILF	755.93 (570.75)	137.33 (162.31)
Left UF	579.18 (607.17)	100.22 (84.91)

## 4. Discussion

We evaluated techniques for uncertainty quantification to provide more accurate and reliable predicted probabilities segmentation outputs applied to DL-based tract segmentation. We show quantitatively (Tables 1, 2 and Figure 1) that stochastic approaches with uncertainty awareness have comparable performance to state-of-the-art methods. Additionally, these uncertainty measures have a positive correlation with tract segmentation errors, which indicates uncertainty is a good measure of reliability for tract segmentation (Figures 4–6). Finally, we demonstrate that calibrated predicted probabilities are more representative of the ground truth volume compared to uncalibrated predicted probabilities (Figure 7).

Similar studies have proposed DL-based tract segmentation. TractSeg (10) is a voxel-based approach introduced for multi-label segmentation using 2D U-Nets to segment 72 tracts. As input to the network, TractSeg uses the 3 major peak directions computed from FODs based on CSD (21). TractSeg has an average Dice of 0.84 on 105 subjects from the HCP dataset which is currently the best multi-label tract segmentation performance achieved. Although the authors use a tri-planar approach, the use of 2D CNNs cannot leverage spatial context between slices (62). Additionally, selecting only the 3 major peaks of the FODs computed using CSD may discard important information contained within the dMRI data.

Neuro4Neuro is another voxel-based approach (17). A 3D U-Net is used to segment 25 tracts with an average Dice of 0.75. As input for the 3D CNN, this approach uses DTI. Although the authors used a large cohort to train their models (>1,000 scans), they segment a small number of tracts. This approach obtained lower Dice for the task compared to TractSeg. One explanation is that DTI only models single fiber populations and cannot resolve complex fiber configurations such as fiber crossings (63), resulting in “poor” segmentation for complex structures (i.e., inferior longitudinal fasciculus) (17). Direct comparisons between Neuro4Neuro and our work were not possible since the code was not publicly available, and their test data was an in-house dataset.

DeepWMA (18) is a fiber-based approach that uses a 2D multi-channel fiber feature descriptor to describe fibers obtained from whole-brain tractography. DeepWMA uses a 2D CNN to classify individual fibers into one of 54 possible WM tracts. This approach generalizes well for scans of independently acquired populations. This method reports a performance comparable to TractSeg, average Dice 0.83, for 34 tracts on the HCP dataset (18). However, for a new given patient, DeepWMA requires preprocessing whole-brain tractography which is a time-consuming and computationally expensive step.

Classifyber is another fiber-based approach (8). Classifyber uses an LR classification model to predict whether individual streamlines belong to a tract of interest. Similar to DeepWMA, Classifyber also has a descriptor to represent a streamline based on a set of features (i.e., spatial position, connectivity, etc). Although the method provides high Dice (≥ 0.80 per tract), Classifyber relies on LR where each tract is treated as a separated classification task and does not address the challenges of multi-label classification. As with DeepWMA, tractography is a preprocessing step that is time-consuming and computationally expensive.

In this study, we used TTD and TTA to model epistemic and aleatoric uncertainty, respectively. We evaluated the combination of both in a Hybrid approach. The aim of this approach is to provide additional information about model and data reliability to help inform clinicians' decision making. The TTA approach has the best performance in terms of Dice for all stochastic approaches, however, the Hybrid approach provides a stronger correlation between structure-wise uncertainty (VVC) and segmentation error (1-Dice), indicating it is a good measure of segmentation reliability (Figure 3). These results are observed more strongly in tracts with complex anatomy that are difficult to segment such as ILF (Figures 5, 6).

We used single-rater ground truth annotations for this study. However, multi-rater annotations could improve the quality of segmentation results (less bias toward a single rater) but would introduce inter-rater variance. In this context, we would expect an increase in uncertainty for areas with high variability among the raters and high certainty in areas with high concordance among the raters.

Calibration was performed per tract using a volume-based approach. Calibrated volume estimates are more likely to encompass the ground truth volume than the uncalibrated volume estimates (Figure 7). Calibration can help to reduce false negatives by allowing the user to select a volume from a larger quantile to err on the side of caution, e.g., during surgery/planning, stochastic approaches can include the segmentation regions with a low likelihood of belonging to the tract to ensure no potential damage will occur even if it is a low probability event. However, calibration is sensitive to the size of the training set (24) and the quality of the ground truth, meaning that tracts with complex anatomy and inter-subject variability, such as the UF tract, may be difficult to calibrate. Additionally, volume as a metric for calibration may not be sufficient for tract segmentation, and other metrics such as a topology-based assessment to describe tract segmentation coverage should be investigated.

There are two key limitations in this study. First, we validated our methods on research data acquired using a single protocol. For clinical data with different acquisition protocols, DL-based methods can output “poor” segmentation and high epistemic uncertainty due to domain shift (64). While domain adaption (65) can overcome low model performance and high uncertainty, we have not investigated this in the current study. Second, we did not validate subjects with pathologies that would distort WM tissue connectivity, which can result in unusual tract shape and location that might lower the Dice of our proposed method. One future avenue of research is to evaluate our approach to subjects with pathologies that distort normal anatomy, such as brain tumors, in a clinical setting.

## 5. Conclusion

In this study, we presented uncertainty awareness for tract segmentation with accurate and reliable predicted probabilities so that clinicians can use it as a safety tool in preoperative neurosurgical planning. Our stochastic approaches, TTD, TTA, and Hybrid, achieved performance comparable to the state-of-the-art methods while outputting measures of uncertainty. We demonstrated a strong positive correlation between segmentation error and structure-wise uncertainty for our stochastic approaches indicating that our output uncertainties are a good measure of reliability for tract segmentation. We confirmed the importance of volume-based calibration in tract segmentation showing an improved ability to measure tract volumes in complex structures compared to uncalibrated approaches. However, other metrics that describe tracts topology could improve calibration results but require further investigation. We focused our analysis on healthy subjects from the HCP dataset. Future validation is required to demonstrate our approach generalizes to datasets acquired at clinical sites and on patients with brain pathologies that distort normal anatomies, such as edemas or tumors.

## Data Availability Statement

The original contributions presented in the study are included in the article material. The Human Connectome Project (HCP) data is a publicly available dataset (https://db.humanconnectome.org) where we used the 105 subjects. Further inquiries can be directed to the corresponding author.

## Author Contributions

OL and RS: conceptualization. OL: software, data curation, validation, formal analysis, investigation, writing—original draft, and visualization. PB and OL: methodology. PB, JC, SO, KA, and RS: writing—review and editing. RS: resources. SO: funding acquisition. KA, RS, and SO: supervision and project administration. All authors contributed to the article and approved the submitted version.

## Funding

This research was funded by the National Institute for Health Research (NIHR) Biomedical Research Centre based at Guy's and St Thomas' NHS Foundation Trust and King's College London and the NIHR Clinical Research Facility. OL was funded by EPSRC Research Council (EPSRC DTP EP/R513064/1). PB was funded by the Wellcome Flagship Programme (WT213038/Z/18/Z) and Wellcome EPSRC CME (WT203148/Z/16/Z).

## Author Disclaimer

The views expressed are those of the author(s) and not necessarily those of the NHS, the NIHR or the Department of Health.

## Conflict of Interest

The authors declare that the research was conducted in the absence of any commercial or financial relationships that could be construed as a potential conflict of interest.

## Publisher's Note

All claims expressed in this article are solely those of the authors and do not necessarily represent those of their affiliated organizations, or those of the publisher, the editors and the reviewers. Any product that may be evaluated in this article, or claim that may be made by its manufacturer, is not guaranteed or endorsed by the publisher.

## Footnotes

^1^ https://zenodo.org/record/1285152#.YDeqj-qnxH4

^2^ https://github.com/OeslleLucena/TractSegmentation
